# A standardized workflow for surveying recombinases expands bacterial genome‐editing capabilities

**DOI:** 10.1111/1751-7915.12846

**Published:** 2017-11-02

**Authors:** Deirdre E. Ricaurte, Esteban Martínez‐García, Ákos Nyerges, Csaba Pál, Víctor de Lorenzo, Tomás Aparicio

**Affiliations:** ^1^ Systems Biology Program Centro Nacional de Biotecnología (CNB‐CSIC) Campus de Cantoblanco Madrid 28049 Spain; ^2^ Synthetic and Systems Biology Unit Institute of Biochemistry Biological Research Centre of the Hungarian Academy of Sciences Szeged H‐6726 Hungary

## Abstract

Bacterial recombineering typically relies on genomic incorporation of synthetic oligonucleotides as mediated by *Escherichia coli* λ phage recombinase β – an occurrence largely limited to enterobacterial strains. While a handful of similar recombinases have been documented, recombineering efficiencies usually fall short of expectations for practical use. In this work, we aimed to find an efficient Recβ homologue demonstrating activity in model soil bacterium *Pseudomonas putida* EM42. To this end, a genus‐wide protein survey was conducted to identify putative recombinase candidates for study. Selected novel proteins were assayed in a standardized test to reveal their ability to introduce the K43T substitution into the *rpsL* gene of *P. putida*. An ERF superfamily protein, here termed Rec2, exhibited activity eightfold greater than that of the previous leading recombinase. To bolster these results, we demonstrated Rec2 ability to enter a range of mutations into the *pyrF* gene of *P. putida* at similar frequencies. Our results not only confirm the utility of Rec2 as a Recβ functional analogue within the *P. putida* model system, but also set a complete workflow for deploying recombineering in other bacterial strains/species. Implications range from genome editing of *P. putida* for metabolic engineering to extended applications within other *Pseudomonads* – and beyond.

## Introduction

In the post‐genomics era, many potential innovations have been hampered by a dearth of rapid, affordable and comprehensive techniques for genetic modification. Fortunately, recombineering is evolving to enable large‐scale genomic editing with a host of applications, ranging from increases in genomic diversity to the optimization of metabolic pathways for biosynthesis (Wang *et al*., 2009; Gallagher *et al*., 2014). Recombineering refers to directed genetic recombination between foreign DNA and endogenous homologies during DNA replication, as mediated by phage‐derived recombinases (Yu *et al*., 2000; Ellis *et al*., 2001; Marinelli *et al*., 2012). Modern recombineering technologies originated in *Escherichia coli*, in which the β, Exo and γ proteins of λ phage were first exploited in tandem to introduce double‐stranded (ds) DNA into the lagging strand of transient replication forks (Murphy, 1998; Yu *et al*., 2000). Ellis *et al*. (2001) simplified this method, demonstrating the ability of the λ phage β protein to independently mediate the genomic annealing and recombination of single‐stranded (ss) mutagenic oligonucleotides. Finally, Wang *et al*. (2009) developed the landmark recombineering protocol known as Multiplex Automated Genomic Engineering (MAGE): a library of synthesized mutagenic ss‐oligonucleotides is introduced into β‐expressing *E. coli*, allowing for rapid, multisite genomic modification that can be cycled to generate vast spaces of genomic diversity. Since its advent, MAGE recombineering has brought about a multitude of advances, including genome‐wide codon replacement, biosynthesis of lycopene and aromatic amino acids, and comprehensive mutant library generation (Wang *et al*., 2009; Isaacs *et al*., 2011; Gallagher *et al*., 2014; Nyerges *et al*., 2016). Unfortunately, β‐recombinase (Recβ) recombineering activity is not well‐conserved in bacterial species outside of the *Enterobacteriaceae* family (Van Kessel and Hatfull, 2008; Binder *et al*., 2013; Lennen *et al*., 2016); this limited conservation is thought to stem from specificity of host–phage recombinase interactions (Sun *et al*., 2015). The apparent species specificity of recombineering systems has spurred a search for functional Recβ analogues in alternative lineages, with limited success in microbes including *Salmonella*,* P. syringae, Corynebacteria*,* Lactobacilli* and *Mycobacteria* (Van Kessel and Hatfull, 2008; Gerlach *et al*., 2009; Swingle *et al*., 2010; Pijkeren and Britton, 2012; Binder *et al*., 2013; Oh and Pijkeren, 2014). While most reports involve the isolation of a single enzyme, a recent protein survey conducted by Datta *et al*. (2008) produced several *rec* genes in *E. coli,* suggesting the utility of a comprehensive approach to recombinase identification within other microorganisms.

The saprotrophic soil bacterium *Pseudomonas putida* is known for its tolerance to a variety of environmental stresses, including but not limited to organic solvents and reactive oxygen species (Poblete‐Castro *et al*., 2012; Nikel *et al*., 2014; Ramos *et al*., 2015). *P. putida* derives this sturdiness from its EDEMP cycle, a series of metabolic pathways that facilitate NAD(P)H production via carbon recycling processes (Nikel *et al*., 2015). An adaptability to a myriad of harsh conditions has made *P. putida* a popular focus of biosynthetic studies aimed at industrial biotransformations as well as soil and water bioremediation (Garmendia *et al*., 2008; Puchałka *et al*., 2008; Poblete‐Castro *et al*., 2012; Loeschcke and Thies, 2015). Unfortunately, the collection of genetic tools available in *Pseudomonads* is limited in scope and, when used in conjunction, is subject to bottlenecks that hinder the generation of a biotechnological chassis (Martínez‐García and de Lorenzo, 2011; Martínez‐García *et al*., 2011, 2014). Efforts at recombineering in *Pseudomonas* species have been attempted with limited results (Liang and Liu, 2010; Swingle *et al*., 2010). One study has isolated a ssDNA annealing recombinase (termed Ssr) demonstrating β‐like activity in *P. putida* (Aparicio *et al*., 2016). Although the Ssr protein demonstrates an ability to catalyse *P. putida* recombineering without off‐target mutagenesis, levels of recombineering are insufficient for large‐scale use.

This study aimed to isolate a recombinase exhibiting activity levels practical for biosynthetic remodelling in *P. putida*. Using the classical ERF, GP2.5, SAK and SAK4 recombinase families as screening references (Lopes *et al*., 2010), we employed a protein sequence similarity search to identify enzymes with potential *rec* capabilities in *Pseudomonads* genomes. Along with RecT_*Psy*_, Recβ and Ssr as controls, we functionally characterized study candidates with a series of standardized assays to assess recombineering activity. This novel biosynthetic workflow allowed us to describe a new set of recombinases demonstrating activity in *P. putida*. Among these, we identified an ERF superfamily ssDNA binding protein, here termed Rec2, that greatly outperforms current *rec* standards in *P. putida*. Our findings support the application of Rec2 towards future recombineering efforts within *P. putida*, as well as potential extensions of the generated search pipeline and *rec* panel to alternative *Pseudomonads* (and other bacteria in general) for industrial, environmental and biomedical purposes.

## Results

### Sequence search reveals novel protein candidates for recombineering study in *P. putida*

**Figure 1:**
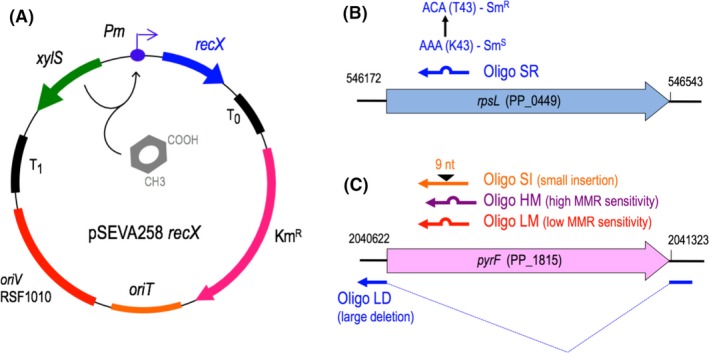
Components of the recombineering testing platform for *Pseudomonas putida* EM42. A. Organization of plasmid series pSEVA258‐*recX* (~8 kb; *recX* indicates cloned recombinase, e.g. *rec1* and *rec2*) for conditional expression of putative recombinases selected for study in *P. putida*, image reproduced from Ref. (Aparicio *et al*., 2016). DNA fragments of all candidate proteins were designed for cloning into the pSEVA258 vector by Gibson assembly, as detailed in *Experimental procedures*. Proper insertion placed all proteins under control of the 3MB‐inducible *Pm* promoter. Additional relevant functional segments include the 3MB‐sensitive regulator *xylS*, flanking transcriptional terminators T_1_ and T_0_, origin of transfer (*oriT*) and replication (*oriV*), and a kanamycin resistance cassette (Km^R^) to enable antibiotic selection of pSEVA258‐*recX*‐transformed cell lines. B. Designation, location and expected change associated with the SR oligonucleotide targeted to the *rpsL* gene of *P. putida*. The gene locus and coordinates are indicated, and the sequence of the SR oligo is listed in Table S1. C. Depicts analogous information for the *pyrF* oligonucleotides employed in this work, reproduced directly from Ref. Aparicio *et al*. (2016).

With its metabolic plasticity and extensive tolerance to environmental stresses, *P. putida* has recently become the focus of many biosynthetic studies related to industrial catalysis and environmental bioremediation (Loeschcke and Thies, 2015). Unfortunately, recombineering capabilities in this organism are limited by the ongoing search for an analogue of *E. coli* λ phage Recβ. Thus, we began this work with a comprehensive sequence survey of *Pseudomonas* sp. genomes for putative functional analogues of the *recβ* gene. Along with *recβ*,* ssr* and *recT*_*Psy*_, we selected ten gene candidates for study in *P. putida* (*rec1* to *rec10*; Table 1), each of which was Gibson‐assembled into the pSEVA258 vector platform (for more information, see *Experimental procedures*). A model of the resulting construct – which harboured the promiscuous origin of replication RSF1010 and the 3‐methylbenzoate (3MB)‐inducible, XylS‐dependent expression promoter *Pm* – is shown in Fig. 1A. Assembled constructs were transformed into *P. putida* EM42, an industrial reference strain stripped of interference factors known to limit heterologous gene expression (Martínez‐García *et al*., 2014).

**Table 1:** Putative ssDNA binding proteins used in this work

Name	Organism	Description (Family)	Length (aa)	Locus tag	Accession no.
Rec1	*P. putida* S16	ERF superfamily single stranded (ss) DNA‐binding protein (ERF)	253	PPS_RS12640	WP_013972384.1
Rec2	*P. putida* CSV86	ERF superfamily ss‐DNA binding protein (ERF)	267	CSV86_RS08085	WP_009397165.1
Rec3	*P. putida* KT2440 Prophage 3	DUF2815 domain‐containing ss‐DNA binding protein (GP2.5)	227	PP_2267	WP_010953240.1
Rec4	*P. sp*. KG01	ss‐DNA binding protein (GP2.5)	232	ACR52_RS27	WP_048731951263.1
Rec5	*P. putida* CSV86	Hypothetical protein (Sak)	243	CSV86_RS06065	WP_009396409.1
Rec6	*P. aeruginosa* phage F116	Hypothetical protein (Sak)	251	F116p19	YP_164283.1
Rec7	*P. aeruginosa* AZPAE14939	Hypothetical protein (Sak)	254	NS55_RS18170	WP_043087219.1
Rec8	*P. putida* UASWS0946	RecT family DNA binding protein (Sak4)	363	QV12_RS02185	WP_043859966.1
Rec9	*P. putida* KB9	DNA binding protein (Sak4)	314	A3K88_RS17255	WP_064314638.1
Rec10	*E. faecalis* V583	RecT family DNA binding protein (Sak4)	307	EF2132	NP_815795.1
RecT_Psy_	*P. syringae* B728a	RecT family DNA binding protein (Redβ‐like)	295	Psyr_2820	AAY37_859.1
Recβ	*E. coli* λ phage	Phage λ ss‐DNA binding protein (Redβ‐like)	261	lambdap84	NP_040617.1
Ssr	*P. putida* DOT‐T1E	ERF family ss‐DNA binding protein (ERF)	252	T1E_1405	AFO47_260.1

### Design of a *rpsL*‐based reporter system in *P. putida*

By inhibiting polypeptide synthesis, streptomycin (Sm) triggers misreads during genomic translation that ultimately lead to bacterial cell death (Ruusala and Kurland, 1984). Several single nucleotide mutations to the *rpsL* gene, which encodes the ribosomal protein S12, have been characterized as conferring Sm resistance in different bacteria (Funatsu and Wittmann, 1972; Timms *et al*., 1992; Gregory *et al*., 2001). This array of modifications make the antibiotic marker a robust tool for genomic mutagenesis in *E. coli* (Jiang *et al*., 2013) and *P. syringae* (Swingle *et al*., 2010). Given the negligible occurrence rates of spontaneous mutants observed in *P. putida* (Jatsenko *et al*., 2010), we chose streptomycin resistance as conferred by oligo‐mediated mutagenesis to the *P. putida rpsL* gene as a measure of putative recombinase activity. To this end, we designed an *rpsL*‐directed ss‐oligonucleotide (SR) whose strand invasion would result in the *rpsL* K43T missense mutation, a *P. putida* analogue of the K42T mutation in *E. coli* (Timms *et al*., 1992). SR was designed to produce a single‐base‐pair mismatch poorly detected by MMR machinery (Babic *et al*., 1996; Kunkel and Erie, 2005); schemata of the K43T mutation and its targeted disruption to *P. putida rpsL* appear in Fig. 1B. In keeping with established strand bias and previous study design (Ellis *et al*., 2001; Costantino and Court, 2003; Aparicio *et al*., 2016), SR was targeted to the lagging strand of the *P. putida rpsL* gene and engineered to evade endogenous MMR activity, in order to maximize putative recombinase efficiency. Further information on the design and synthesis of the mutagenic oligonucleotide can be found in the Experimental procedures section.

### *rpsL*‐directed recombineering assay in *P. putida* EM42 highlights two protein candidates for additional study

**Figure 2:**
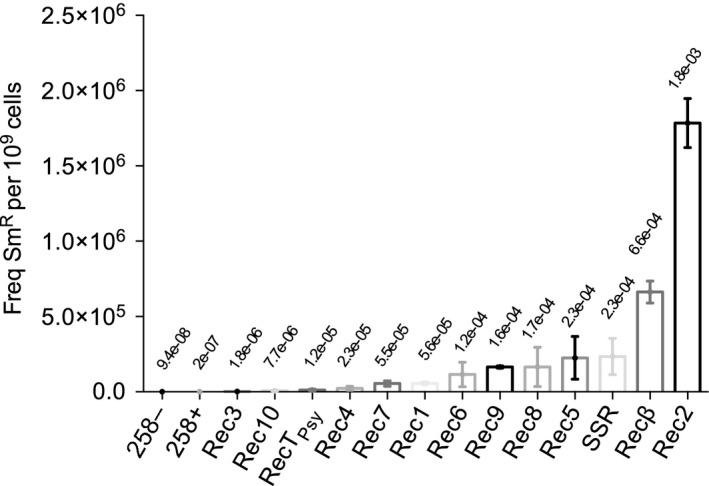
ERF superfamily protein Rec2 exhibits improved recombination frequencies after electroporation with *rpsL*‐directed mutagenic oligonucleotide SR. *Pseudomonas putida* EM42 strains containing pSEVA258 and pSEVA258‐*recX* constructs were induced with 3MB before electroporation with mutagenic oligo SR. After overnight recovery in TB, culture dilutions were plated on LB and LB‐Sm solid media. Colony counts were determined, and correct SR‐mediated changes were checked by amplifying and sequencing the *rpsL* gene from 5 to 10 Sm^R^ clones associated with each experiment (see *Experimental procedures* for further details). Recombineering efficiency was calculated as the number of Sm^R^ mutants per 10^9^ cells. Column graphs of the resulting data were generated with graphpad prism. Column values represent mean recombineering frequencies for each experimental condition; standard error (SE) bars are also shown. Each data set represents at least two independent experimental replicates. Recombineering values are shown on a linear scale to facilitate frequency comparison. Absolute frequency values (mutants per viable cell) are shown above each column. Controls 258^+^ and 258^−^ indicate Sm^R^ colonies arising from *P. putida* EM42 (pSEVA258) cells electroporated or not, respectively, with the mutagenic oligonucleotide.

To measure the ability of candidate recombinases to direct oligo‐mediated mutagenesis during genomic replication, *P. putida* EM42/pSEVA258‐*recX* strains and *P. putida* EM42 containing the empty vector pSEVA258 were grown to mid‐logarithmic phase and treated with 3MB to induce protein expression. Competent cell mixtures were electroporated with the SR oligonucleotide and allowed to recover overnight before selective plating on LB and LB‐Sm solid agar media. An empty vector strain shocked without the SR oligo was included to measure the frequency of spontaneous Sm resistance in *P. putida*. Average recombineering frequencies and standard error values for each protein candidate, calculated from absolute colony counts, are shown in Fig. 2. Five to ten Sm^R^ clones derived from each experimental condition were isolated, PCR amplified and sequenced at the targeted *rpsL* region to verify acquisition of the proper K43T mutation.

We first noted that Sm^R^ mutants were detected at low levels in *P. putida* EM42 (pSEVA258) control strain whether the bacteria were electroporated with the SR oligonucleotide or not. In both cases, absolute recombineering frequencies (RF) were around 10^−7^ mutants/viable cell (Fig. 2). One hundred percent of resistant colonies arising from the electroporated strain harbored the proper *rpsL* mutation. This detection of the K43T mutation in the pSEVA258‐containing control strain thus indicates the ability of the SR oligonucleotide to mutate *rpsL* in the absence of an exogenously expressed recombinase. Similar background levels of recombineering activity in *P. putida* have been observed previously (Aparicio *et al*., 2016). In contrast, the Sm^R^ clones stemming from cells that were not electroporated with the SR oligonucleotide did not bear the K43T change. These Sm^R^ colonies indicate the spontaneous occurrence of Sm resistance mutations, a phenomenon already reported in *E. coli* (Timms *et al*., 1992).

Each protein candidate exhibited levels of recombineering activity surpassing experimental controls (RF > 10^−7^). Sequencing verified the acquired K43T mutation in all colonies originating from protein‐expressing strains. As shown in Fig. 2, allelic exchange rates were diverse, ranging from 1.8 × 10^−6^ (Rec3) to 1.8 × 10^−3^ (Rec2). A single recombinase family did not appear to be superior in recombineering activity, although Rec3 and Rec4 of the GP2.5 family produced the lowest recombineering values of proteins derived from the *Pseudomonas* genus. The lowest efficiency of all proteins tested was detected in Rec3, a recombinase identified in prophage 3 of *P. putida* KT2440 (Martínez‐García *et al*., 2015) – a prophage that has been eliminated from the EM42 strain. Rec10, a protein derived from the gram‐positive *E. faecalis* species, produced relatively efficient recombineering levels (7.7 × 10^−6^), with RecT_*Psy*_ from *P. syringae* performing only slightly better (1.2 × 10^−5^). Rec6 of *P. aeruginosa* emerged as a high outlier among the non‐*P. putida* proteins, producing recombineering frequencies comparable to Ssr and superior to *P. putida* Rec1. While several recombinases producing superior RF scores came from the *P. putida* species (Rec2, Rec5, Rec8 and Rec9), average recombineering frequencies of all but one of these enzymes were equal to or lower than those of the current Ssr standard.

*Pseudomonas putida* EM42 cultures expressing the ERF family ss‐binding protein Rec2 and, surprisingly, the λ phage Recβ protein exhibited respective average recombineering frequencies of 1.8 × 10^−3^ and 6.6 × 10^−4^. These were the only values surpassing those of Ssr‐expressing strains (RF = 2.3 × 10^−4^). With recombineering frequencies from three to eight times greater than those of Ssr, Recβ and Rec2 were selected as promising candidates for further investigation.

### Growth curve assays reveal slight toxicity of the Rec2 recombinase

**Figure 3:**
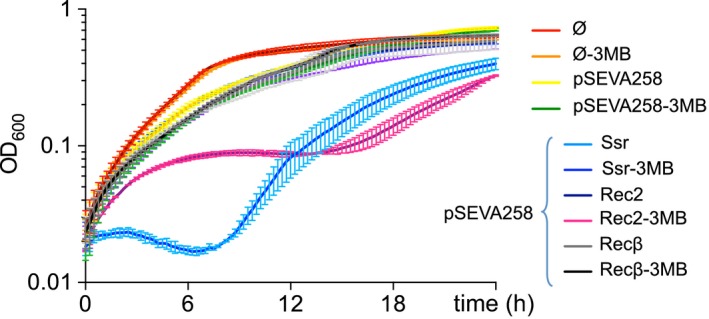
Liquid growth profiles demonstrate slight toxicity of the Rec2 protein. To measure the effects of protein toxicity on cell growth, curves were generated for 3MB‐induced and non‐induced experimental strains using a Spectramax M2e Microplate Reader (Molecular Devices). We prepared overnight LB‐Km cultures of *Pseudomonas putida* EM42 strains containing pSEVA258‐*rec2*, pSEVA258‐*ssr* and pSEVA258‐*recβ*, as well as EM42/pSEVA258 and EM42 controls. Overnight cultures were back‐diluted in LB‐Km with and without 3MB inducer to an initial OD_600_ = 0.05 before being loaded into a 96‐well microtiter plate. Liquid growth profiles of 1 mM 3MB‐induced (*t* = 0) and non‐induced strains were measured over a 24 h period (interval *t* = 15 min). Time elapsed (h) is shown on the *x*‐axis, while optical density at 600 nm (OD_600_) is shown on the *y*‐axis. Strain type is indicated in the figure legend. Individual points represent mean values, and horizontal brackets indicate SE calculated from two independent experimental replicates.

The known toxicity of Ssr – and concomitant associated stress on *P. putida* growth – led us to wonder whether Rec2 and Recβ might show similar toxicity profiles (Aparicio *et al*., 2016). Thus, before continuing with recombineering tests, we generated growth profiles for *P. putida* EM42 strains containing the *ssr*,* recβ* and *rec2* genes, along with controls, in the presence and in the absence of the 3MB inducer (see Experimental Procedures for details). Results appear in Fig. 3. While all strains exhibited similar growth profiles in the absence of the inducer, strains expressing Ssr and Rec2 were heavily impacted by the introduction of 3MB. Ssr induction resulted in negative *P. putida* growth rates during the first seven hours of the experimental window, with an average minimum OD_600_ ~ 0.015 being observed at *t* = 6.5 h (Fig. 3, Ssr, blue line). The impact of induction on Rec2‐expressing strains was less severe. Strains exhibited lower, but non‐negative early growth rates, with optical densities plateauing at an average OD_600_ ~ 0.09 at *t* = 7 h (Fig. 3, Rec2, pink line). These results indicate moderate toxicity in Rec2, as compared to the high capacity of growth inhibition exhibited by Ssr. No growth difference indicating toxicity was detected between 3MB‐induced and non‐induced strains containing *recβ* (Fig. 3, Recβ, grey and black lines overlap with growth profiles of control strains).

### The efficiency of Rec2 is conserved in effecting the E50* mutation in pyrF

Successful genetic remodelling of bacterial chasses using recombineering relies on the capacity to mediate mutagenesis at a range of genetic sites, independent of gene function. Given the efficiency of Rec2 and Recβ in altering the *rpsL* gene of *P. putida*, we extended our exploration of recombinase activity to site‐directed editing of an alternative genetic marker: the *pyrF* gene of *P. putida*. Briefly, *pyrF* disruptions in *P. putida* yield cells both auxotrophic for uracil and resistant to 5FOA, a toxic analogue of uracil (O'Donovan and Neuhard, 1970; Galvao and de Lorenzo, 2005). Scientists have long exploited the dual nature of this gene to isolate edited cell lines in assays of site‐directed mutagenesis (Boeke *et al*., 1984; Galvao and de Lorenzo, 2005). Thus, following protocols developed for *P. putida* EM42 in a previous study (Aparicio *et al*., 2016), we conducted a second round of recombineering experiments with Rec2 and Recβ using the *pyrF‐*directed LM oligonucleotide (Table S1); the Ssr protein was also tested as a control. The LM oligonucleotide was designed to introduce an A‐G mismatch that replaced the E50 residue of *pyrF* with a stop codon (Aparicio *et al*., 2016). The LM and SR mutagenic oligonucleotides are equivalent in nature of mutation and levels of MMR detection. The details of the LM oligonucleotide and its site‐directed mutagenesis of *pyrF* are depicted in Fig. 1C. A detailed description of *pyrF*‐directed recombineering procedures, including LM design and synthesis, can be found in the Experimental Procedures section.

**Figure 4:**
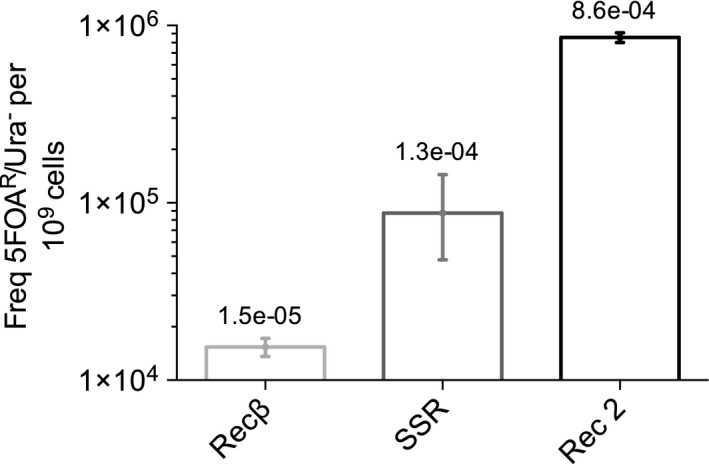
Rec2 maintains recombineering efficiency in entering the LM mutation into the *pyrF* gene of *Pseudomonas putida*. *P. putida* EM42 strains containing pSEVA258‐*ssr*, pSEVA258‐*recβ* and pSEVA258‐*rec2* were induced with 3MB before electroporation with the LM oligonucleotide (Table S1). After a 6 h recovery period, culture dilutions were plated on selective M9‐Cit‐Ura and M9‐Cit‐Ura‐5FOA solid media. Fifty 5FOA^R^ clones associated with each recombinase were replicated on M9‐Cit and M9‐Cit‐Ura‐5FOA solid media to distinguish authentic *pyrF* mutants (Ura auxotrophs, no growth on M9‐Cit) from spontaneous 5FOA^R^ colonies (Ura heterotrophs, growth on M9‐Cit). Colony counts were determined and recombineering efficiency calculated (number of authentic *pyrF* mutants ‐5FOA^R^/Ura^−^ CFU/10^9^ viable cells). Column graphs of the resulting data were generated with graphpad prism as described in Fig. 2. Each data set represents at least two independent experimental replicates. Recombineering values are shown on a logarithmic scale. Absolute frequency values (mutants per viable cell) are shown above each column.

Mid‐logarithmic cultures of *P. putida* EM42 expressing Rec2, Recβ and Ssr were made competent before electroporation with the LM oligonucleotide; after recovery, appropriate dilutions were plated on M9‐citrate solid media supplemented with Ura (viable cells) or Ura/5FOA (allelic changes), and colony counts were corrected by estimating occurrence of spontaneous, non‐*pyrF*‐related mutants (growing on M9‐citrate). The results of these experiments can be seen in Fig. 4. Rec2 produced an average RF value of 8.6 × 10^−4^, over seven times that of the Ssr protein (RF = 1.3 × 10^−4^). Notably, although levels of Rec2 recombineering activity were lowered in *pyrF*‐directed experiments compared to *rpsL*, the magnitude of increase in RF relative to Ssr was maintained. Recβ produced the lowest levels of recombinase activity, with an average RF = 1.5 × 10^−5^. Due to its poor performance under the *pyrF* reporter system, we decided to exclude Recβ from further recombineering experiments. The discrepancy in Recβ performance between *P. putida* reporter systems will be addressed in the Discussion.

While Recβ activity was not conserved within the *pyrF*‐directed system, Rec2 performed at least seven times better than Ssr in both recombineering settings. Further, growth assays indicated diminished toxicity in Rec2 as compared to Ssr in *P. putida* EM42. These promising features led us to conduct a final round of experiments with the ERF family ssDNA binding protein Rec2, testing its ability to enter a range of mutations into the *P. putida* genome.

### Rec2 demonstrates efficiency in effecting a range of mutations in the *pyrF* gene

**Figure 5:**
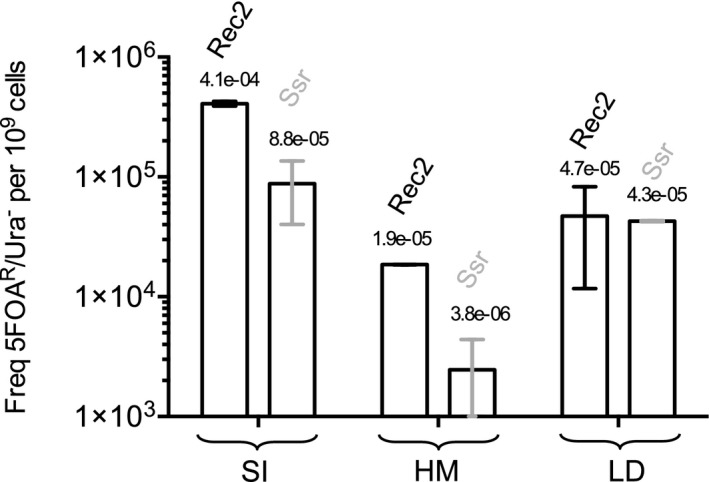
Rec2 is able to enter a range of mutations in *Pseudomonas putida*. *P. putida* EM42 strains containing pSEVA258‐*ssr* and pSEVA258‐*rec2* were assayed as explained above for *pyrF* mutagenesis (see Fig. 4 legend) but using the following *pyrF*‐directed oligonucleotides (Table S1): SI (introduces a 9‐nt insertion of three stop codons), HM (generates a mismatch highly detectable by MMR, creating a stop codon) and LD (mediates the complete deletion of *pyrF*). Each data set represents at least two independent experimental replicates. Column graphs of the resulting data were generated with graphpad prism as described in Fig. 2 **.** Recombineering values are shown on a logarithmic scale.

In a final set of recombineering assays, we employed Rec2 to introduce a range of genomic edits to the *pyrF* gene. As detailed in a previous study (Aparicio *et al*., 2016), the LD, SI and HM oligonucleotides were designed and coupled with Ssr in *P. putida* to enter different types of mutations into *pyrF*: respectively, a complete gene deletion (702‐nt); a short insertion (9 nt) of three stop codons; and a missense mutation of K55 into a stop codon, involving an A‐A mismatch highly detectable by MMR machinery. Thus, we conducted a round of analogous *pyrF*‐directed recombineering experiments in *P. putida* with Rec2, testing the Ssr protein in parallel to confirm the robustness of results. The average RF values produced by Rec2 and Ssr using the various *pyrF*‐directed oligonucleotides are shown in Fig. 5. As in LM‐mediated experiments, Rec2 recombineering assays conducted with SI and HM produced RF values folds greater than those of the Ssr protein. Consistent with the previous study (Aparicio *et al*., 2016), SI‐mediated experiments involving Rec2 produced the greatest recombineering frequencies, with an average RF = 4.1 × 10^−4^. HM‐mediated Rec2 experiments produced the lowest average RF = 1.9 × 10^−5^, likely due to interference by MMR‐repair machinery. LD‐mediated Rec2 experiments yielded intermediate results, with an average RF = 4.7 × 10^−5^. In comparison, Ssr‐driven recombineering assays produced inferior values for SI and HM oligonucleotides (8.8 × 10^−5^ and 3.8 × 10^−6^ respectively) but similar to those with the LD sequence (4.3 × 10^−5^; see below). In summary, improved recombineering frequencies of Rec2 were upheld across a variety of *pyrF*‐directed experiments, indicating the utility of this recombinase in effecting a broad range of mutations in the *P. putida* genome.

## Discussion

Our comprehensive sequence similarity search for recombinogenic proteins within *Pseudomonas* and associated bacteriophage genomes allowed us to identify novel recombinases with potential activity in *P. putida*. We also included in this study three previously documented recombinases: Recβ from *E. coli*, RecT_*Psy*_ from *P. syringae* and EF2132 from *E. faecalis* (here designated Rec10). Under the *rpsL* reporter platform, all assayed *recs* demonstrated some degree of recombineering activity, encompassing three orders of magnitude between the least (Rec3) and the most active proteins (Rec2). While allelic exchange rates varied, all candidates showed high specificity in promoting oligonucleotide‐mediated mutagenesis. Most *recs* originated from phage‐related genomic regions of the *P. putida* genus; however, proteins derived from more distant relatives *P. aeruginosa* (Rec6 and Rec7) and *P. syringae* (RecT_*Psy*_), and even from unrelated species *E. coli* (Recβ) and *E. faecalis* (Rec10), demonstrated appreciable activity in *P. putida* EM42. Further, upon completion of *rpsL*‐directed assays, no association could be found between recombineering activity and either recombinase family or species proximity to *P. putida*. Previous recombineering attempts in environmental and pathogenic bacteria have largely relied on proteins originating from the host species or close relatives; this approach has led to successful editing in *Mycobacterium tuberculosis* (Van Kessel and Hatfull, 2008), *Lactobacillus reuteri* and *Lactococcus lactis* (Pijkeren and Britton, 2012), *Pseudomonas syringae* (Swingle *et al*., 2010) and *Bacillus subtilis* (Sun *et al*., 2015). Our work challenges assumptions of species specificity based on these reports, instead suggesting promiscuity of oligonucleotide‐mediated recombinase activity. Such non‐specific activity has been documented in other bacteria, as in the case of *Corynebacterium glutamicum* (Binder *et al*., 2013) and *E. coli* (Datta *et al*., 2008). While the first study focused only on RecT of Rac prophage, in the second, authors detected high rates of recombineering activity in *E. coli* from an array of recombinases originating from both gram‐negative and gram‐positive bacteria (10^−4^ to 10^−1^ mutants/viable cell). While such rates of allelic exchange reflect an increase in two orders of magnitude from the numbers we have observed (10^−6^ to 10^−3^ mutants/viable cell), we note the use of an *E. coli* ∆*mutS* strain devoid of mismatch repair machinery, which could have increased recombineering rates by >100‐fold (Costantino and Court, 2003). In contrast, *P. putida* EM42 – a streamlined derivative of *P. putida* KT2440 (Martínez‐García *et al*., 2014) – contains a fully intact MMR system, making a direct, quantitative study comparison difficult.

In terms of recombineering efficiency, we identified several novel proteins performing at rates approaching the *P. putida* Ssr standard (Aparicio *et al*., 2016). *P. putida* proteins Rec5 (Sak family), Rec8 and Rec9 (Sak4 family) all produced approximate recombineering frequencies of 10^−4^ mutants/viable cell within the EM42 strain. Interestingly, despite its distant relation to *P. putida*, Rec6 of *P. aeruginosa* (Sak4 family) catalysed recombineering at similar rates (10^−4^ mutants/viable cell). Among recombinases producing poorer results, it should be noted that a low outlier emerged in the only *rec* gene derived from the *P. putid*a KT2440 genome: *rec3* (GP2.5 family, 10^−7^ mutants/viable cell). With rates of ~10^−5^ mutants/viable cell, RecT_Psy_ of *P. syringae* (Redβ‐like family) and Rec10 of *E. faecalis* (Sak4 family) also fell short of Ssr standards. These results deviate from higher efficiencies reported of each protein in *P. syringae* (Swingle *et al*., 2010) and *E. coli* (Datta *et al*., 2008) respectively. Only two recombinases outperformed the Ssr protein: ERF family protein Rec2 (*P. putida* CSV86) and Recβ (*E. coli* λ phage). Both proteins yielded recombineering values ~10^−3^ mutants/viable cell, with the former exhibiting rates three times those of the latter. Despite the concept of a broad host spectrum for phage‐derived recombinases supported by this and other studies (Datta *et al*., 2008), the impressive performance of Recβ in *P. putida* was unexpected. Recβ has only been shown to catalyze bacterial genomic recombination of foreign ssDNA in enterobacteriaceae (Nyerges *et al*., 2016). In contrast, it fails to function in species such as *P. syringae* (Swingle *et al*., 2010) and *C. glutamicum* (Binder *et al*., 2013). Recβ activity in *P. putida* KT2440 has been documented at ~10^−7^ mutants/viable cell (Luo *et al*., 2016), a result equal to, if not lower than, basal recombineering rates in this organism. Notwithstanding our use of an alternative plasmid/reporter system, we take note of Recβ activity reaching ~10^−3^ mutants/viable cell, a value nearly four orders of magnitude greater than the one reported above.

To check the robustness of the above results, we probed the ability of promising candidates Rec2 and Recβ to direct oligo‐mediated mutagenesis of an alternative reporter gene: *pyrF*. While the *pyrF* reporter system facilitates selection of allelic changes resulting in gene disruption, it contains an inherent drawback: Ura‐auxotrophic *pyrF* knockouts grow slower than wild‐type Ura heterotrophs on LB‐rich media supplemented with uracil (Fig. S1). In the context of recombineering experiments, such a growth differential can lead to bias in strain representation post‐electroporation; over time, quickly dividing wild‐type cells attain higher frequencies in the population, while the global representation of slower‐growing mutants decreases. This trend results in an underestimation of mutant rates that is aggravated by longer recovery times, a phenomenon observed in early trials of *pyrF*‐directed recombineering (data not shown). To limit this bias, we restricted recovery periods within the *pyrF* reporter system to 6 h (Aparicio *et al*., 2016), whereas *rpsL* reporter assays (unaffected by this trend) were simplified with overnight recovery times. As seen in Fig. 4, Rec2 and Recβ successfully mediated recombination in *P. putida* under the *pyrF* reporter system, with Rec2 once again surpassing Recβ. However, despite protocol adaptation, we observed a discrepancy in Recβ performance between the *rpsL* and *pyrF* reporter platforms: while the differential recombineering rate was just three times in favour of Rec2 under *rpsL*, under the *pyrF* reporter, Rec2 showed sixty times the amount of activity exhibited by Recβ. We argue that the mild toxicity of Rec2 led to slower growth of Rec2‐expressing cultures after electroporation. Rec2 toxicity, for which a mechanism of action is currently unknown, could lead to a lag phase or a higher death rate in Rec2‐expressing cultures during post‐shock recovery. Slower Rec2 culture growth would yield a lower growth differential between wild‐type and mutant cells during this period, as compared to Recβ cultures. As explained above, a greater growth differential in Recβ cultures would aggravate the underestimation of final mutational rates, accounting for diminished Recβ performance under the *pyrF* reporter system. This said, although Recβ showed noticeable recombineering activities under both experimental conditions, Rec2 alone outperformed Ssr under both reporter platforms within *P. putida*, making it our sole candidate for further recombineering study.

The main experimental body of this work was conducted by introducing single‐base mutations poorly detected by the MMR system into the *rpsL* and *pyrF* genes of *P. putida*. However, large‐scale genomic‐editing projects often necessitate the introduction of a variety of mutations into the target genome, including insertions, deletions and, depending on the desired modification, changes sensitive to endogenous repair machinery. For this reason, we concluded this study with an assay of Rec2 ability to enter an array of changes into the *pyrF* gene. As seen in Fig. 5, Rec2 consistently outperformed Ssr in introducing insertions and MMR‐sensitive point mutations to the *pyrF* gene. However, both proteins produced full *pyrF* deletions at similar rates. While the reason for this result is unknown, we highlight the difference in toxicity observed between Ssr and Rec2 post‐induction (Fig. 3). The impact of this disparity on growth rate can be appreciated in post‐recovery optical densities, taken six hours after culture back‐dilution to OD_600_ ~ 0.1: Rec2 produced an average OD_600_ ~ 0.4, while Ssr produced OD_600_ values equal to or below 0.1. As mentioned above, higher growth rates exacerbate the underestimation of mutant representation associated with the *pyrF* reporter platform. Thus, although we cannot exclude interference by Rec2‐specific recombineering factors (e.g. optimal induction time, length of mutagenic oligonucleotides and protein–oligonucleotide interactions), we suspect the relatively worse Rec2 recombineering efficiencies under the *pyrF* system can be attributed to a greater growth differential between wild‐type and mutant cells during the post‐electroporation recovery period.

In summary, we have isolated an ERF family protein capable of catalyzing mutagenic recombination in *P. putida* with high efficiency, in some cases surpassing previous standards by an order of magnitude. While Rec2 performs at levels below those of Recβ in *E. coli* (Wang *et al*., 2009; Nyerges *et al*., 2016), we note that the frequencies reported in this study reflect a single cycle of recombineering. We expect that optimized protocols involving several cycles would produce results closer to those seen with Recβ in *E. coli*. Further, the *P. putida* EM42 cell factory, while purged of certain endonucleases (Martínez‐García *et al*., 2014) that could inhibit strand displacement (Mosberg *et al*., 2012), harbours a fully functional MMR system. As mentioned, MMR machinery is known to interfere with the MAGE platform in various bacterial species, dampening final rates of mutagenesis. For this reason, experimental set‐ups aimed to facilitate deep genome engineering usually rely on permanent or transient suppression of MMR (Wang *et al*., 2009; Gallagher *et al*., 2014; Lennen *et al*., 2016; Nyerges *et al*., 2016). We expect that implementation of the same approach in *P. putida* would greatly enhance Rec2 performance, facilitating for projects based on the deployment of high levels of combinatorial genomic diversity.

In practical terms, the suite of recombinases identified in this study has broader implications. With protocols adjusted, Rec2 offers the potential to facilitate auspicious biosynthetic ventures in *P. putida* ‐ for example, the rewiring of metabolic pathways and implantation of genetic and metabolic circuits in cell factory strains. We note that the array of proteins tested in this study was standardized to the broad‐host‐range plasmid RSF1010. Given the promiscuity of this testing platform, we contend that the broad spectrum of recombinase constructs tested contains viable candidates for future study in environmental bacteria beyond *P. putida* – in *Pseudomonas aeruginosa* and other gram‐negative bacteria, for example. Given its remarkable performance in *P. putida*, Rec2 becomes a particularly promising candidate for future studies in its parent strain. Once perfected, deep genetic programming in *Pseudomonas* and other environmental bacteria could unlock radical advances in biocatalysis and bioremediation, extending to targeted purification of contaminated soil reserves and biosynthesis of biomedical compounds of interest.

## Experimental procedures

### Bacterial strains and growth conditions

Table 2 lists the strains and plasmids employed in this work. Experimental strains were grown in liquid Luria Bertani (LB) (10 g l^−1^ tryptone, 5 g l^−1^ yeast extract and 5 g l^−1^ NaCl) or glycerol‐free terrific broth (TB) (24 g l^−1^ yeast extract, 12 g l^−1^ tryptone, 9.4 g l^−1^ K_2_HPO_4_, 2 g l^−1^ KH_2_PO_4_) with shaking (170 rpm) at 30°C (*P. putida*) or 37°C (*E. coli*). M9 minimal media (Sambrook *et al*., 1989) was supplemented, when stated, with 0.2% w/v citrate for *P. putida* growth. Solid media were prepared at the ratios listed above, with 15 g l^−1^ agar added. When necessary, liquid and solid media were supplemented with 20 μg ml^−1^ of Uracil (Ura), 50 μg ml^−1^ of kanamycin (Km), 50 μg ml^−1^ of chloramphenicol (Cm), 100 μg ml^−1^ of streptomycin (Sm) or 250 μg ml^−1^ of 5‐fluoroorotic acid (5‐FOA). During recombineering experiments, 1 mM of 3‐methylbenzoate (3MB) was added to mid‐logarithmic‐phase cultures of *P. putida* EM42 to induce protein expression for 30 min.

**Table 2:** Bacterial strains and plasmids used in this work

Strain or plasmid	Relevant characteristics^a^	Reference
*Strains*		
*Escherichia coli* CC118	Cloning host; Δ*(ara‐leu) araD* Δ*lacX174 galE galK phoA thiE1 rpsE* (Sp^R^) *rpoB* (Rif^R^) *argE*(Am) *recA1*	Grant *et al*. (1990)
*Escherichia coli* HB101	Helper strain used for conjugation; F^−^ λ^−^ *hsdS20*(rB^−^ mB^−^) *recA13 leuB6(*Am*) araC14* Δ(*gpt‐proA*)62 *lacY1 galK2*(Oc) *xyl‐5 mtl‐1 thiE1 rpsL20*(Sm^R^) *glnX44*(AS)	Boyer and Roulland‐Dussoix (1969)
*Pseudomonas putida* EM42	KT2440 derivative; Δprophage1 Δprophage4 Δprophage3 Δprophage2 ΔTn*7* Δ*endA‐1* Δ*endA‐2* Δ*hsdRMS* Δflagellum ΔTn*4652*	Martínez‐García *et al*. (2015)
*Plasmids*		
pRK600	Helper plasmid used for conjugation; *oriV*(ColE1), RK2 (*mob*+ *tra*+); Cm^R^	Kessler *et al*. (1992)
pSEVA258	Cloning vector; *oriV* RSF1010); *(xylS‐Pm;* standard multiple cloning site; Km^R^	Silva‐Rocha *et al*. (2013)
pSEVA258‐*rec*X	pSEVA258 derivative bearing X recombinase indicated in Table 2; *oriV* (RFS1010); cargo [*xylS‐Pm → recX*], Km^R^	This work

a Antibiotic markers: Sp, spectinomycin; Rif, rifampicin; Sm, streptomycin; Cm, chloramphenicol; Km, kanamycin.

### General DNA manipulation, DataBank Search, Plasmid Construction and Oligonucleotide design

Standard DNA manipulations were conducted according to manufacturer's recommendations and previously established protocol (Sambrook *et al*., 1989). Isothermal/Gibson assembly was carried out according to procedures outlined in (Gibson *et al*., 2009; Gibson, 2011). ssDNA binding proteins with potential recombineering activity were selected from the NCBI non‐redundant (nr) protein sequence database: drawing from a recent survey of bacteriophage genomes for homologues to Rad52, Rad51 and Gp2.5 superfamily recombinases (Lopes *et al*., 2010), homologues from the major recombinase families were identified on *Pseudomonas* phage genomes. We designated the following proteins belonging to ERF, GP2.5, Sak and Sak4 families as sequence references for a subsequent sequence similarity search: ERF recombinase from *P. aeruginosa* phage D3 (GenBank no. NP_061548); GP2.5 recombinase from *P. putida* gh‐1 phage (GenBank no. NP_813756); SAK recombinase from *P. aeruginosa* phage F116 (GenBank no. YP_164283) and SAK4 recombinase from *P. aeruginosa* phage 73 (GenBank no. YP_001293439). We conducted a search for sequence similarity using the National Center for Biotechnology Information (NCBI) protein–protein BLAST tool (http://blast.ncbi.nlm.nih.gov). A BlastP search on the NCBI nr protein sequence database against *P. putida* and related bacteriophage genomes produced seven proteins bearing high sequence similarities to reference recombinases (Recs1, 2, 3, 5, 6, 8 and 9). An equivalent search of all *Pseudomonas* and related bacteriophage genomes using GP2.5 and SAK phage recombinase references – from *Pseudomonas* phages gh‐1 and F116 respectively – produced two hypothetical recombinases also included in this study (Recs4, 7). Finally, the EF2132 recombinase RecT of *Enterococcus faecalis* has been described to facilitate efficient recombineering in a phylogenetically distant gram‐negative host (Datta *et al*., 2008). Therefore, we also tested EF2132 (here termed Rec10) for recombineering functions in *Pseudomonas putida*. In total, we selected ten novel protein candidates for investigation. We also incorporated three recombinases previously tested for activity in *Pseudomonas*. Due to its reported levels of activity in *P. syringae* (Swingle *et al*., 2010), we selected RecT_Psy_, a homologue of the *E. coli* Rac prophage recombinase RecT. Finally, we included the original *E. coli λ* phage recombinase Recβ*,* which has been shown to demonstrate low levels of activity in *P. putida* KT2440 (Luo *et al*., 2016). As a control, we added to our study the current leading candidate for recombineering in *P. putida*, the T1E_1405 protein of *P. putida* DOT‐T1E (named Ssr). Ssr has been shown to mediate lagging strand invasion and recombination in *P. putida* at low frequencies with high specificity of mutagenesis (Aparicio *et al*., 2016). Table 1 characterizes all protein candidates tested in this work. The open reading frames (ORFs) for putative recombinases 1, 2, 4, 5, 6, 7, 8, 9 and 10 were synthesized *de novo* by GeneCust (GeneCust Europe, Luxembourg) with flanking sequences to allow cloning into the pSEVA258 vector. Following the experimental design outlined in (Aparicio *et al*., 2016), the 5′ flanking sequence (5′‐*TGGAGTCATGACCATGCCTAGGCCGCGGCCGCGCgaattc*AGA AGGAGAATATACC‐3′) contained (i) a 40‐bp homology to the sequence upstream of the EcoRI site in pSEVA258 (italics, EcoRI in lowercase), (ii) a ribosome binding site (underlined), and (iii) a 7‐bp spacer. Downstream of the stop codon, the 3′ flanking sequence (5′‐ggatccTCTAGAGTCGACCTGCAGGCATGCAAGCTTGCGG‐3′) contained a 40‐bp homology to the sequence downstream of the BamHI site (lowercase) in pSEVA258. The *rec3* gene was PCR amplified directly from the KT2440 chromosome using primers Rec3FW/Rec3REV. The *recT*_*Psy*_ and *recβ* genes were PCR amplified from the pUCP24 (Swingle *et al*., 2010) and pKD46 (Datsenko and Wanner, 2000) plasmids with primer pairs RecT_Psy_FW/RecT_Psy_REV and RecβFW/RecβRV respectively. All primer pairs listed (Table S1) harbored sequence tails containing the same features described for Recs1‐10. In this way, all *rec* genes were placed in a standard expression platform of pSEVA258 just as the *ssr* gene (Aparicio *et al*., 2016). *rec* genes, either from gene synthesis or from PCR amplification, were Gibson‐assembled into EcoRI/BamHI‐digested pSEVA258 and electroporated into *E. coli* CC118 (Table 2). Transformed constructs were checked for proper insertion by sequencing with primers 238F and PS2 (Table S1). Constructs were finally introduced into *P. putida* EM42 via tripartite mating with helper strain HB101 (Table 2), as outlined in Ref. Martínez‐García and de Lorenzo (2012). Plasmid‐containing strains were selected for on M9‐Citrate‐Km solid media. As an additional check, individual clones of *P. putida* EM42/pSEVA258‐*recX* (where X is the recombinase specimen 1–10) were replicated on the same media, and plasmid constructs isolated by miniprep were digested with EcoRI/BamHI to verify the transformants. Control strains of *P. putida* EM42 harboring pSEVA258 and pSEVA258‐*ssr* were constructed in a previous work (Aparicio *et al*., 2016). Oligonucleotides used in this work were provided by Sigma‐Aldrich (St Louis, MO, USA) and are characterized in Table S1. *pyrF‐*directed oligonucleotides employed in genomic recombineering experiments (namely, LM, HM, SI and LD) were described previously in Aparicio *et al*. (2016). Using the same design criteria applied for the LM oligonucleotide (length, 5′ phosphorothioate protection, folding energy, MMR System sensitivity, etc.), the SR oligonucleotide was modelled after the *rpsL* gene of *P. putida* KT2440*,* which encodes the ribosomal protein S12. SR generates an A‐G mismatch (poorly detected by MMR) that introduces a single nucleotide change (A→C) in codon K43 (AAA‐Lys), resulting in T43 (ACA‐Thr). The K43T missense mutation is analogous to the classical K42T mutation of the *rpsL* gene of *E. coli* (Timms *et al*., 1992; Jiang *et al*., 2013), which confers resistance to streptomycin.

### Assays for recombineering efficiency in *P. putida* EM42

The ability of recombinase candidates to incorporate foreign ss‐oligonucleotides into endogenous DNA forks occurring during genetic replication (Ogawa and Okazaki, 1980) was first measured through acquisition of Sm resistance by *P. putida* strains electroporated with *rpsL*‐targeted SR (Table S1). In *rpsL*‐directed recombineering experiments, *P. putida* EM42/pSEVA258‐*recX* strains were incubated overnight in LB‐Km at 30°C with shaking (170 rpm). A strain transformed with empty vector pSEVA258 was included as an additional control. Overnight strains were back‐diluted to OD_600_ = 0.1 in 20 ml of fresh LB‐Km and regrown in identical conditions to OD_600_ = 0.4–0.5. Cultures were supplemented with 1 mM 3MB and incubated for an additional 30 min to induce the expression of Rec proteins. Competent cell preparation was then performed at RT by pelleting cells at 3220 *g* for 5 minutes, washing successively with 10, 5 and 1 ml of 300 mM sucrose and finally resuspending in 200 μl of the same solution. One hundred microlitres of aliquots of prepared cells was then supplemented with 1 μl of 100 μM SR oligonucleotide stock (~3 μg). Well‐mixed cultures were transferred into 2‐mm gap width cuvettes (Bio‐Rad Laboratories, Hercules, CA, USA) before electroporation at 2.5 kV with a Micropulser^TM^ apparatus (Bio‐Rad Laboratories). *P. putida* EM42/pSEVA258 control cultures were electroporated with and without oligo SR to determine background recombineering levels. Samples were immediately inoculated in 5 ml of fresh TB and allowed to recover overnight at 30°C with shaking (170 rpm). Recovered cell cultures were plated at appropriate dilutions on LB‐Sm and LB solid media and incubated 24 hours at 30°C. After absolute colony counts were taken, the recombination frequency (RF) was calculated as the ratio between Sm‐resistant cells and viable cells (cells growing on LB), and normalized to 10^9^ viable cells. To ensure accuracy of allelic changes, primers rpsL‐Fw and rpsL‐Rv were used to perform colony PCR on 5‐10 clones derived from each recombinase; the obtained 0.8 Kb fragment (encompassing the *rpsL* gene) was sequenced with primer rpsL‐Fw (Table S1). Recombineering activity of the most promising protein candidates (RF>2.3 × 10^−4^) was assayed in a different reporter platform (*pyrF*) to verify the robustness of *rpsL*‐targeted recombineering results. *pyrF*‐targeted recombineering experiments were conducted according to methods described in Ref. (Aparicio *et al*., 2016). The LM oligonucleotide (Table S1) was used in a first set of experiments to introduce a stop codon into the *pyrF* gene. Recombineering frequencies were calculated by plating dilutions on M9‐Citrate‐Uracil (viable cells) vs. M9‐Citrate‐Ura‐5‐FOA (allelic changes). In a second set of experiments, the most active Rec protein was characterized, in comparison with Ssr, using the HM, LD and SI oligonucleotides (Table S1). These oligonucleotides each introduce a different genomic change in *pyrF* as explained above. For all *pyrF* experiments, recombineering frequency was calculated as the ratio between 5FOA^R^/Ura‐auxotrophic cells and total viable cells (cells growing on M9‐Cit‐Ura), and normalized to 10^9^ viable cells. To distinguish authentic *pyrF*‐mutant colonies from spontaneous 5FOA^R^ mutants, fifty 5FOA^R^ clones of each recombinase were replicated on M9‐Cit/M9‐Cit‐Ura‐5FOA plates as outlined in Ref. (Aparicio *et al*., 2016). Colonies growing on both media were discounted as spontaneous *pyrF*‐unrelated mutants. Frequencies were multiplied by the ratio between verified *pyrF* mutants (cells growing on M9‐Cit‐Ura‐FOA only) and total # 5FOA^R^ CFU (cells growing on all media).

### Additional experiments

To determine the effect of protein toxicity on strain growth, profiles were generated for 3MB induced and non‐induced experimental strains using a Spectramax M2e Microplate Reader (Molecular Devices, Sunnyvale, CA, USA). Cultures of *P. putida* EM42 strains harbouring pSEVA258‐*rec2*, pSEVA258‐*ssr* and pSEVA258‐*recβ*, as well as EM42/pSEVA258 and EM42 controls, were grown overnight in LB‐Km‐shaken liquid media at 30°C. Overnight strains were back‐diluted to OD_600_ = 0.05 on fresh LB‐Km medium before being loaded into a 96‐well microtiter plate. Liquid growth rates of 1 mM 3MB‐induced (*t* = 0) and non‐induced strains were measured over a 24 h period (interval *t* = 15 min) following the OD_600_. Two biological replicates were carried out for each experimental condition.

## Conflict of interest

None declared.

## Supporting information


**Table S1.** Oligonucleotides used in this work.
**Fig S1.** Growth of *P. putida* EM42*∆pyrF*.
