# Supplementary material for: A standardized workflow for surveying recombinases expands bacterial genome‐editing capabilities
Source: Microb Biotechnol. 2017 Nov 2;11(1):176–88. doi: 10.1111/1751-7915.12846 (PMC5743808; doi:10.1111/1751-7915.12846)
Supplement: Supplementary file 1 — Table S1. Oligonucleotides used in this work. Fig S1. Growth of P. putida EM42∆pyrF. [file MBT2-11-176-s001.pdf]

Supplementary Materials

TABLE S1. Oligonucleotides used in this work.

Oligo	Sequence 5'→3' [b)]	Genetic Modification/Use	Reference
SR	G*T*C*A*GACGCACA CGGCATACTTTACGC AGTGCCGAGTTAGGT TTTgTCGGCGTGGTG GTGTACACACGGGTG CACACGCCACGACGC TGC	Change of K43 (AAA) into T43 (ACA) in *rpsL* gene confers streptomycin resistance. Mismatch A-G (low MMR sensitivity).	This work
LM	C*T*T*G*AGGTCCAG GAACACTTCGAAGCC CTTGTCACACAGGGTT TaGACAATGCCCGAA GCGCTGCTGGTGAAC AGCTCCT TGCCAA*C *C*T*T	Change of E50 (GAA) into a stop codon (TAA) in *pyrF* gene. Mismatch A-G (low MMR sensitivity)	Aparicio et al (2016)
HM	G*G*G*A*ATGTCGTG GAACTTGAGGTCCAG GAACACTTCGAAGCC CTaGTCACACAGGGT TTCGACAATGCCCGA AGCGCTGCTGGTGA* A*C*A*G	Change of K55 (AAG) into a stop codon (TAG) in *pyrF* gene. Mismatch A-A (high MMR sensitivity)	Aparicio et al (2016)
SI	C*T*T*G*AGGTCCAGG AACACTTCGAAGCCC TTGTCACACAGGGTc tattatcaTTCGACAA TGCCCGAAGCGCTGC TGGTGAACAGCTCCTT GCCAA*C*C*T*T	Insertion of three stop codons (9 bp) in *pyrF* (short insertion)	Aparicio et al (2016)
LD	A*C*A*G*GCATCGGTG GTTCGGCACAGGCCC TTGCTGGACAGCCGC AGGTTAAGGGCAGGG TCTCTTGGCAAGTCGA AAACGGCGCGCATTG TAAACGAAGTG	Complete deletion of *pyrF* (long deletion)	Aparicio et al (2016)

Rec3FW	TGGAGTCATGACCATGCCTAGGCCGCGGCCGCGCGAATTC*AGAAGGAGA*ATATACC**ATG**TCCTATCAGAAACGCCC	With Rec3REV, to amplify the *rec3* gene for Gibson assembly.	This work
Rec3REV	CCGCAAGCTTGCATGCCTGCAGGTCGACTCTAGAGGATCC**TTA**GAAGTCTTCTTCGTAAGTG	With Rec3REV, to amplify the *rec3* gene for Gibson assembly.	This work
RecβFW	TGGAGTCATGACCATGCCTAGGCCGCGGCCGCGCGAATTC*AGAAGGAGA*ATATACC**ATG**AGTACTGCACTCGCAAC	With RecβREV, to amplify the *recβ* gene for Gibson assembly.	This work
RecβREV	CCGCAAGCTTGCATGCCTGCAGGTCGACTCTAGAGGATCC**TCA**TGCTGCCACCTTCTG	With RecβFW, to amplify the *recβ* gene for Gibson assembly.	This work
RecTFW	TGGAGTCATGACCATGCCTAGGCCGCGGCCGCGCGAATTC*AGAAGGAGA*ATATACC**ATG**TCCGCAAGAAACGTTG	With RecTREV, to amplify the $recT_{Psy}$ gene for Gibson assembly.	This work
RecTREV	CCGCAAGCTTGCATGCCTGCAGGTCGACTCTAGAGGATCC**TCA**TGCGGTTTCTCCG	With RecTREV, to amplify the $recT_{Psy}$ gene for Gibson assembly.	This work
238F	GGTTTGATAGGGATAAGTCCAG	With PS2, to conduct diagnostic PCR and sequencing of *rec* insertions in pSEVA258	This work
PS2	GCGGCAACCGAGCGTTC	With PS2, to conduct diagnostic PCR and sequencing of *rec* insertions in pSEVA258	Aparicio et al (2016)
rpsL-Fw	GACATGAAATGTTGCCGATG	With rpsL-Rv, to amplify *rpsL* gene of *P. putida*	This work

		(0.8 Kb)	
rpsL-Rv	CTGTTCTTGCGTGCTTTGAC	With rpsL-Fw, to amplify *rpsL* gene of *P. putida* (0.8 Kb)	This work

b) Asterisks denote phosphorothioate linkages. Nucleotide changes mediated by oligos are shown in lower case. In the sequences of oligos used for Gibson assembly, EcoRI and BamHI sequences are underlined, positions of synthetic ribosomal binding sites appear in italics and start/stop codons are shown in bold face.

REFERENCES

Aparicio, T., Jensen, S.I., Nielsen, A.T., de Lorenzo, V. and Martínez-García, E. (2016) The Ssr protein (T1E_1405) from *Pseudomonas putida* DOT-T1E enables oligonucleotide-based recombineering in platform strain *P. putida* EM42. *Biotechnol J* **11**: 1309-1319.

Fig S1. Growth of *P. putida* EM42Δ*pyrF*

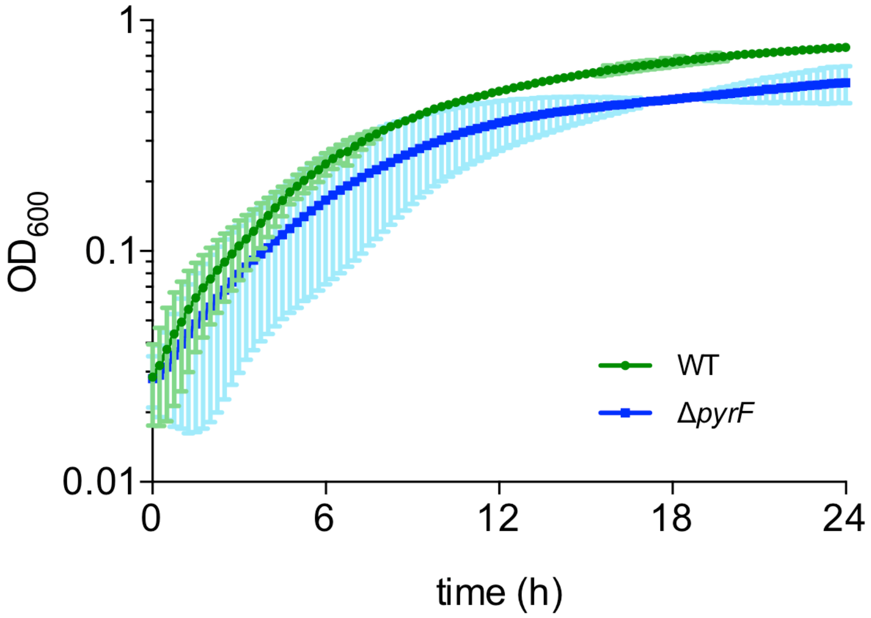

To demonstrate the effects of a complete deletion of the *pyrF* gene on *P. putida* EM42 growth, curves were generated for wild-type *P. putida* EM42 and a Δ*pyrF* strain (1). Optical densities of shaken liquid strains were taken over a period of 24 hours using a Spectramax M2e Microplate reader (Molecular Devices, CA, USA). We prepared overnight LB-Ura cultures of *P. putida* EM42 wild-type and Δ*pyrF* strains at 30˚ C. Overnight strains were back-diluted in LB-Ura to an initial OD_{600} = 0.03 before being loaded into a 96-well microtiter plate. Liquid growth rates were measured over a 24 h period (interval t = 15 min). Time elapsed (h) is shown on the x-axis, while optical density at 600nm (OD600) is shown on the y-axis. Strain type is indicated in the figure legend. Individual points represent mean values and horizontal brackets indicate SE calculated from two independent experimental replicates.
